# Primary health care among rural pregnant women in China: achievements and challenges in maternal mortality ratio

**DOI:** 10.1017/S1463423619000306

**Published:** 2019-06-25

**Authors:** Le Yang, Hongman Wang

**Affiliations:** The School of Health Humanities, Peking University, 38 Xueyuan Road, Haidian, Beijing, China

**Keywords:** the maternal mortality ratio, China, primary health care, health policy

## Abstract

**Background:**

The maternal mortality ratio (MMR) is not only an important indicator of maternal and infant safety, but also a sign of the development of economy, education, and medical care in a country. In the last 60 years, the Chinese government has implemented various strategies and policies to reduce the MMR, especially in the rural areas.

**Aim:**

This study aimed to discuss the strategies developed by the Chinese government, showing the successful experience of Chinese intervention programs and highlighting the challenges to the government in the context of current economic and social status.

**Method:**

This study probed into the Chinese government’s efforts and achievements in the MMR reducing by reviewing the relevant health policies, extracting the data from *China Health Statistics Yearbook of 2015*, analyzing the reduction of maternal death in rural areas and the major causes from 1991 to 2015, comparing the MMR trend in urban and rural areas, and discussing the changes of the situation in China.

**Finding:**

Although it seems that Chinese government’s efforts have brought evangel to the rural pregnant women and significantly reduced rural maternal mortality, the government still needs to develop more equitable and flexible primary health care policies to narrow the imbalance in health resource allocation and pay more attention to the health care for the rural-to-urban migration in China.

## Introduction

The maternal mortality ratio (MMR) is defined as the annual number of maternal deaths divided by the total number of live births and then multiplied by 100 000. The MMR could easily inform the risk of maternal death per pregnancy or per birth; however, its implications attract more attention of the policymakers and scholars. The MMR is not only an important indicator of maternal and infant safety, but also a sign of the development of economy, education, and medical care in a country (Cross *et al.*, 2010).

The global maternal mortality is unacceptably high and approximately 830 women die from pregnancy or childbirth-related complications around the world every day. It was estimated that in 2015, roughly 303 000 women died during and following pregnancy and childbirth (WHO, 2016). The high number of maternal deaths in some areas of the world reflects the inequality in access to health services and highlights the gap between the rich and poor (Yang *et al.*, 2014). Compared with those in rich regions, women in poor regions are more likely to develop the MMR due to lack of health knowledge, limited medical resources, and poor pregnancy conditions. Almost all maternal deaths (99%) occur in developing countries (Hogan *et al.*, 2010), and most of them could have been prevented (Alkema *et al.*, 2016). There are large disparities not only between countries, but also within countries, and between women with high and low income and between women living in rural and urban areas (WHO, 2016), especially in China, the rapid economic development of economy has caused large disparities between people with high and low income and between people living in rural and urban areas (Du *et al.*, 2009; Gao *et al.*, 2009).

Reducing the MMR came into focus when it became one of the eight Millennium Development Goals (MDGs), with the goal of reducing the MMR by three quarters from 1990 to 2015 (Sachs and McArthur, 2005). Most developing countries have struggled to reduce MMR, including China. The Chinese Government has implemented many national and local programs to reduce disparities in access to prenatal services and make more affordable and effective treatment available through developing training programs for health workers and implementing health care policies and monitoring systems for pregnant women. Various strategies have been carried out in China over the last 60 years to improve the maternal and child health care (Wang *et al.*, 2008) and have made remarkable achievements. Hence, it is significant to show the experience of Chinese MMR reduction measures and furthermore to discuss the challenges to the government with the hope that the government could develop more effective and equitable policies to improve the primary health care among rural pregnant women.

### Challenges of reducing maternal mortality in China

The MMR is higher in rural areas and in communities with lower income, less education and poorer health care (You *et al.*, 2012). There are so many reasons causing the high MMR, maternal death in China is related to biomedical, reproductive, health service, socioeconomic and cultural factors (Du *et al.*, 2009; Harris *et al.*, 2010; You *et al.*, 2012; Liang *et al.*, 2011). Most of them could be prevented by the government’s intervention in financial investment, human resources, and health care education. China is a developing country with around 1.4 billion population, representing approximately 18% of the world’s population. Therefore, it is a big challenge for the government to allocate medical and health care resources purely fair and equitable.

In China, it is common and well known that there is a large gap between the urban and the rural areas, and there is no exception in the health care. 80% of health care resources have been allocated to urban areas, especially to the metropolis (Luo *et al.*, 2007; Huang *et al.*, 2010). Without enough, necessary, and effective health care during pregnancy in rural areas, the MMR among rural pregnant women once has become the main cause for lowering the whole health status. It has been shown that in China, 80% of maternal deaths were farmers, 58.5% of whom delivered at home and 35.5% died of childbirth at home (Liang *et al.*, 2007). It is really urgent to solve the problem of backward maternal health care in rural areas of China.

There is also a noticeable gap between eastern and western China, and the western rural areas are worse. In 2000, the MMR was 21.2 and 114.9 per 100 000 live births in eastern and western China, respectively. In 2013, the gap between eastern and western China was narrower, to be more specific, 14.6 versus 33.5 per 100 000 live births. However, the MMR of some remote and poor provinces of western China remains stably high. For example, the MMR of Tibet was 70.7 per 100 000 live births, which was significantly higher than those in other provinces, and 62 per 100 000 live births in Qinghai (Chen *et al.*, 2015 **)**. Even though the government has invested a large amount of resources, the primary health care service in these areas is still weak.

Chinese primary health care system is not perfect in terms of quality; therefore, people feel reluctant to seek health care (Cheng, *et al.*, 2017), thus increasing the risk of MMR in rural areas. In the current situation of rural China, that is, the economic development is relatively backward, and people’s living conditions are generally poor, the rural pregnant women’s safe and effective delivery at home is not available for they could not have sanitary and good environment during the process.

The midwifery profession plays a crucial role in the health care system, especially in the maternal and child health care system. However, as China does not report legislation recognizing midwifery as an autonomous regulated profession (Castro *et al.*, 2016) and most midwives have low educational level and only receive 3-year training in non-university health schools, the midwives receive very little professional recognition in Chinese hospital-based system. China does not have formal midwives (Gao *et al.*, 2017), midwives are more commonly regarded as obstetric nurses, rather than professional technicians who can monitor the labor process and complete the delivery independently. Midwives are not professionally recognized in the Chinese health system in terms of their education or employment, which leads to overuse of caesarean section and other interventions (Wiklund *et al.*, 2018). In China, the midwives have been running the risk of being marginalized. They lack the opportunities to further their education, thus failing to polish and update their skills to keep current, which causes the continuous degradation of midwifery techniques and a huge gap in midwifery care.

Furthermore, the health workforce distribution in China is not perfectly equitable, which shows the disparities between the urban and the rural as well as between the east and west China.

### Initiatives to reduce MMR to meet MDGs

**Figure 1.**
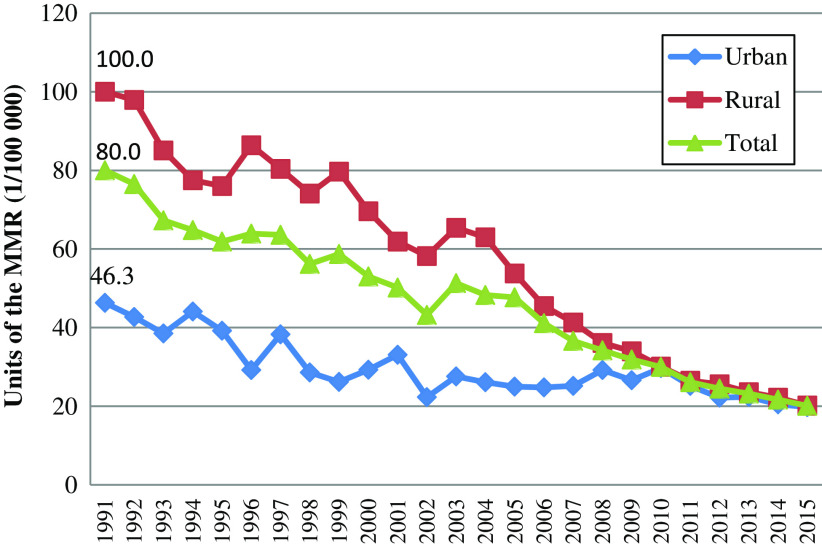
The maternal mortality ratio in China from 1991 to 2015.

Source: National Health and Family Planning Commission of the People’s Republic of China 2016: *China Health Statistics Yearbook of 2015* (In Chinese).

In accordance with MDGs to reduce the MMR by three quarters from 1990 to 2015, the State Council Committee for Women and Children together with the Ministry of Health and the Ministry of Finance have implemented an intervention, which is called ‘reducing maternal mortality and eliminating neonatal tetanus’ (‘Reducing and Eliminating’) since 2000 (Department of Maternal and Child Health and Community Health, 2005). The program has specifically focused on the remote and rural areas in mid-western with a high or very high burden of maternal mortality. The program improved the rate of delivery in hospital among rural pregnant women, and the whole MMR has been slashed from 80 per 100 000 live births in 1991 to 20.1 per 100 000 live births in 2015 (Figure 1). (National Health and Family Planning Commission of the People’s Republic of China, 2016).

Apart from that, the government implemented a project to eliminate neonatal tetanus in 378 counties of 12 provinces/autonomous regions/municipalities in central and western China, since 2000 (Liang *et al.*, 2012). The project aimed to reduce the cost of health care for labor and birth for poor families, established an emergency obstetric service (called Green Channel) and an emergency aid center, developed training programs for obstetrics and gynecology personnel and pediatric staff, built a high-quality health care system, reduced charges for prenatal, delivery, and postnatal care, and offered health education (Yang *et al.*, 2014).

As part of the recent rural health care system reform, the government increased the subsidy for hospitalized delivery and improved women’s health status in the rural areas. From 2009, the government has paid the subsidy of 500 RMB for hospitalized delivery among rural pregnant women. During 2009–2011, the government has invested 7.9 billion RMB for the hospitalized delivery among 27.266 million rural pregnant women. In addition, the government provided the free prepregnancy health services for 12.05 million rural pregnant women, such as the free health education, checkups, risk assessment, and consultation, with the coverage of 96.5% in 2015. Furthermore, the new cooperative medical scheme, established from 2003, is a heavily subsidized voluntary health insurance program aiming at reducing the risk of catastrophic health spending for rural residents in China (You and Kobayashi, 2009), which greatly relieved the economic burden of hospitalized delivery of rural pregnant women and increased the standard and safe pregnancy among rural areas.

### Methods of evaluating initiatives

The data used in this study was based on the Maternal Mortality Surveillance Network of China. Actually, the report of maternal death in China has been completed over time. The Maternal Mortality Surveillance Network of China was established in 1989. From 1990 to 1995, it covered 247 monitoring districts (countries) in 30 provinces, autonomous regions and municipalities in the inner land of China, and from 2007 to the present, it has been extending to 336 monitoring districts (countries). The number of maternal deaths in the monitoring districts (county) was monitored by a three-level health care network; all the data was collected and reported level by level. The data in this paper was extracted from *China Health Statistics Yearbook of 2015*, and the major causes of maternal death and the reduction in different times and regions from 1991 to 2015 were analyzed.

## Results

With the strengthening of primary health care among rural pregnant women, the gap between the urban and the rural areas in the MMR has been greatly narrowed. In 1991, the MMR of urban areas was 46.7 per 100 000 live births versus 100 per 100 000 live births in rural areas, but in 2015, the MMR of urban areas was 19.8 per 100 000 live births versus 20.2 per 100 000 live births in rural areas (Figure 1), which showed the remarkable achievements in the control of MMR in rural areas.

**Figure 2.**
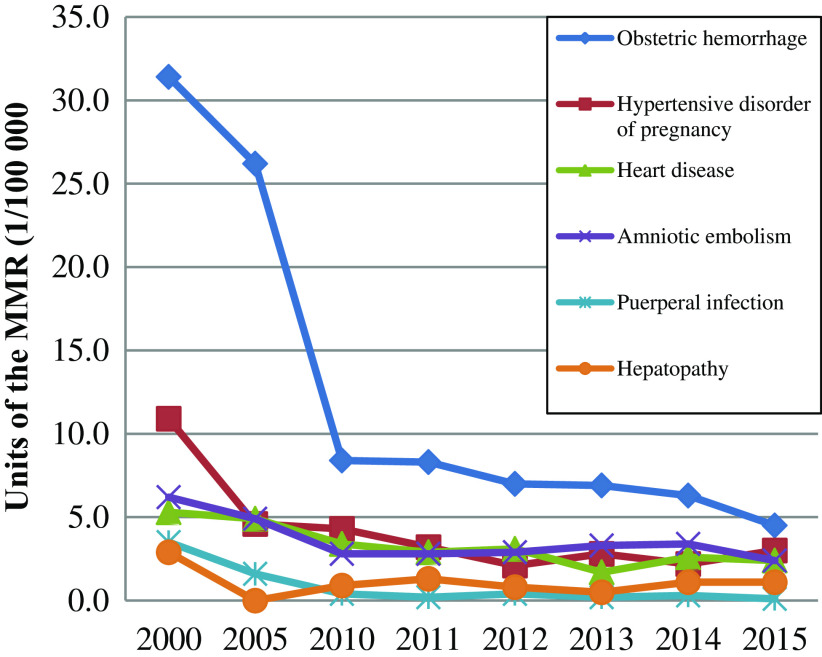
The main causes of maternal mortality ratio in rural areas of China from 2000 to 2015.

Source: National Health and Family Planning Commission of the People’s Republic of China 2016: *China Health Statistics Yearbook of 2015 and 2013* (In Chinese).

Meanwhile, the main causes of MMR in rural areas have been controlled and managed effectively (Figure 2), especially the obstetric hemorrhage and hypertensive disorder of pregnancy.

The decline in the maternal mortality in rural areas is due to the strong economic investment of the Chinese government in the maternal and neonatal care in remote and rural areas, the new cooperative medical scheme for pregnancy, the strengthening systemic care during pregnancy, the effective implementation of the ‘Reducing and Eliminating’ project and the substantial increase in hospital delivery rates. The increase in the rate of hospital delivery, prenatal checkup and education, and postpartum visit has effectively led to the effective control of obstetric hemorrhage caused by improper operation of the traditional rural delivery method.

### Discussion and way forward for the future

With efforts in the last 60 years, China has made outstanding progress in the MMR reduction, especially in rural areas, and accumulated precious experiences for other developing countries. However, the MMR in developing countries in 2015 was 239 per 100 000 live births versus 12 per 100 000 live births in developed countries (WHO, 2016). Compared with developed countries, China still has many jobs to do. The lack of medical resources in rural and remote China and the education and retention of human resources related to obstetrics continue to be important challenges in China. Meanwhile, there are still some new challenges emerging.

The new situation of rural-to-urban migration, which has made a tremendous contribution to national economic development and families’ income, causes problems in the management of pregnant women and the primary health care service supply. In Shanghai, the total number of live births of floating population has increased from 16 458 in 1996 to 63 968 in 2015, and the average MMR of Shanghai declined from 28.84 per 100 000 live births in 1996 to 25.57 per 100 000 live births in 2005, among which the MMR of local pregnant women showed a dramatic decrease from 22.47 per 100 000 live births in 1996 to 1.64 per 100 000 live births in 2005, while the MMR of migrant pregnant women showed a slight decrease but were still high, which was 54.68 per 100 000 live births in 1996 and 48.46 per 100 000 live births in 2005 **(**Zhu *et al.*, 2007 **)**.

In 2013, the Third Plenary Session of the 18^th^ Central Committee of the Communist Party of China decided to start the ‘two-child fertility policy’ where either the husband or the wife from a single-child family, that is, Chinese couples with one spouse being an only child, would be permitted to have two children. Since January 1, 2016, the country officially launched a new family planning policy for allowing one couple to have two children, which is the ‘universal two-child policy’. Because of the large population base and the implementation of the universal two-child policy, which changes the conventional one-child policy implemented for decades from the 1980s, the pressure of MMR control is heavier in the coming wave of fertility.

Furthermore, China has a very high rate of caesarean section (CS) between 2008 and 2014, and the overall annual rate of cesarean deliveries increased to 34.9% in 2014 in China (Betran *et al.*, 2016; Li *et al.*, 2017). CS is known to lead to an increase in the risk of maternal mortality and carries risks in subsequent pregnancies for the health effects caused by CS, such as uterine rupture, abnormal placentation, ectopic pregnancy, stillbirth, and preterm birth for women (Sandall *et al.*, 2018). The increasing cesarean deliveries would bring an additional new challenge to the control of maternal mortality and morbidity in the context of the ‘universal two-child policy’.

Political will and commitment played a key role in reducing maternal deaths nationwide (Liang *et al.*, 2012). The remarkable achievements of the intervention of MMR in rural areas of China show that the effective health policy and strong investment are of great importance to improve the health status in China. Policymakers are expected to establish more equitable and effective health policies to strengthen the primary health care service for the pregnant women, improve the hospitalized delivery, increase the number of health workers in rural areas, enhance the accessibility of health care for rural pregnant women at home, standardize and regulate the implementation of cesarean section, and provide standard health service and reduce the risk in pregnancy.

## Financial of support

The study was supported by the Major Program of Beijing Municipal Social Science Fund (No. 17ZDA16).
